# *RYR2* Mutations Are Associated With Benign Epilepsy of Childhood With Centrotemporal Spikes With or Without Arrhythmia

**DOI:** 10.3389/fnins.2021.629610

**Published:** 2021-04-07

**Authors:** Mei-Gang Ma, Xiao-Rong Liu, Yuan Wu, Jie Wang, Bing-Mei Li, Yi-Wu Shi, Tao Su, Bin Li, De-Tian Liu, Yong-Hong Yi, Wei-Ping Liao

**Affiliations:** ^1^Institute of Neuroscience and Department of Neurology of the Second Affiliated Hospital of Guangzhou Medical University, Key Laboratory of Neurogenetics and Channelopathies of Guangdong Province and the Ministry of Education of China, Guangzhou, China; ^2^Department of Neurology, The First Affiliated Hospital of Guangxi Medical University, Nanning, China

**Keywords:** *RYR2*, mutations, epilepsy, BECTS, arrhythmia

## Abstract

*RYR2* encodes ryanodine receptor 2 protein (RYR-2) that is mainly located on endoplasmic reticulum membrane and regulates intracellular calcium concentration. The RYR-2 protein is ubiquitously distributed and highly expressed in the heart and brain. Previous studies have identified the *RYR2* mutations in the etiology of arrhythmogenic right ventricular dysplasia 2 and catecholaminergic polymorphic ventricular tachycardia. However, the relationship between *RYR2* gene and epilepsy is not determined. In this study, we screened for novel genetic variants in a group of 292 cases (families) with benign epilepsy of childhood with centrotemporal spikes (BECTS) by trio-based whole-exome sequencing. *RYR2* mutations were identified in five cases with BECTS, including one heterozygous frameshift mutation (c.14361dup/p.Arg4790Pro fs^∗^6), two heterozygous missense mutations (c.2353G > A/p.Asp785Asn and c.8574G > A/p.Met2858Ile), and two pairs of compound heterozygous mutations (c.4652A > G/p.Asn1551Ser and c.11693T > C/p.Ile3898Thr, c.7469T > C/p.Val2490Ala and c.12770G > A/p.Arg4257Gln, respectively). Asp785Asn was a *de novo* missense mutation. All the missense mutations were suggested to be damaging by at least three web-based prediction tools. These mutations do not present or at low minor allele frequency in gnomAD database and present statistically higher frequency in the cohort of BECTS than in the control populations of gnomAD. Asp785Asn, Asn1551Ser, and Ile3898Thr were predicted to affect hydrogen bonds with surrounding amino acids. Three affected individuals had arrhythmia (sinus arrhythmia and occasional atrial premature). The two probands with compound heterozygous missense mutations presented mild cardiac structural abnormalities. Strong evidence from ClinGen Clinical Validity Framework suggested an association between *RYR2* variants and epilepsy. This study suggests that *RYR2* gene is potentially a candidate pathogenic gene of BECTS. More attention should be paid to epilepsy patients with *RYR2* mutations, which were associated with arrhythmia and sudden unexpected death in previous reports.

## Introduction

*RYR2* gene (OMIM^∗^180902) resides on chr1q43. It encodes ryanodine receptor 2 protein (RYR-2) that is mainly located on endoplasmic reticulum (ER) membrane. RYR-2, as a subunit of homotetramer, constituted the calcium channel that regulates intracellular calcium concentration. The RYR-2 protein is ubiquitously distributed and highly expressed in the heart and brain, during all stages of human development (Martin et al., 1998). The *RYR2* mutations have been established in the etiology of arrhythmogenic right ventricular dysplasia 2 (ARVD2) and catecholaminergic polymorphic ventricular tachycardia (CPVT) (Tiso et al., 2002). A recent study identified an *RYR2* mutation in a family with CPVT, in which an affected individual presented epilepsy (Yap and Smyth, 2019). However, the relationship between *RYR2* gene and epilepsy is not determined.

Benign epilepsy of childhood with centrotemporal spikes (BECTS), also known as Rolandic epilepsy (OMIM^#^ 245570)^1^, is a common form of epilepsy in children and estimated to affect approximately 0.2% of the population (Kramer et al., 2002). Although BECTS is generally regarded as autosomal dominant inheritance with age-dependent penetrance, the genetic causes of BECTS are largely undetermined. Recent studies have shown that several genes, including *GRIN2A*, *ELP4*, *SRPX2*, and *DEPDC5* (Lemke et al., 2013; Lal et al., 2014; Reinthaler et al., 2014), are associated with BECTS. Approximately 4.9% of the patients with BECTS harbor a variant of the glutamate ionotropic receptor *N*-methyl-D-aspartate type subunit 2A (*GRIN2A*) gene (Lemke et al., 2013). In this study, whole-exome sequencing (WES) was performed on a group of patients with BECTS and their parents (trios). *RYR2* mutations, including two compound heterozygous missense mutations, were identified in five unrelated BECTS cases with or without arrhythmia. This study suggests that *RYR2* gene is potentially a candidate pathogenic gene of BECTS. More attention should be paid to epilepsy patients with *RYR2* mutations, which were associated with arrhythmia and sudden unexpected death in previous reports (Bagnall et al., 2016).

## Materials and Methods

### Patients

A cohort of 292 cases with BECTS was recruited. The patients were from the Epilepsy Center of the Second Affiliated Hospital of Guangzhou Medical University between July 2016 and February 2020.

Clinical characteristics of the affected individuals were collected, including present age, gender, age at seizure onset, seizure course, detailed family history, general and neurological examination results, and effective antiepileptic drugs (AEDs). Magnetic resonance imaging (MRI) scans were performed to detect any brain structure abnormalities. Long-term (24 h) video electroencephalogram (EEG) examination was obtained that included open–close eyes test, intermittent photic stimulation, hyperventilation, and sleeping recording. The results were reviewed by two qualified electroencephalographers. Epileptic seizures and epilepsies were diagnosed according to the criteria of the Commission on Classification and Terminology of the ILAE (1981, 1989, 2001, 2010, and 2017). All of the subjects were followed up for at least 1 year in the epilepsy center.

The studies adhered to the guidelines of the International Committee of Medical Journal Editors with regard to patient consent for research or participation and received approval from the ethics committee of the Second Affiliated Hospital of Guangzhou Medical University. All participants gave written informed consents.

### Whole-Exome Sequencing

Blood samples were obtained from the probands, their parents, and available family members to determine the origin of the identified genetic variants. Genomic DNA was extracted from peripheral blood using a QuickGene DNA whole-blood kit (Fujifilm, Tokyo, Japan). Trio-based WES was conducted on the Illumina HiSeq 2500/4000 platform by BGI-Shenzhen (Shenzhen, China) as previously reported (Wang et al., 2018; Shi et al., 2019). To identify candidate causative variants in each trio, we adopted a case-by-case analytical approach. We first prioritized the rare variants with a minor allele frequency (MAF) <0.005 in the 1000 Genomes Projects, Exome Aggregation Consortium, and gnomAD. We retained potentially pathogenic variants containing frameshift, nonsense, canonical splice site, initiation codon, and missense mutations predicted as being damaging by *in silico* tools^2^. We then screened the potential disease-causing mutations in each case under five models: (1) epilepsy-associated gene mutations; (2) *de novo* dominant mutations; (3) autosomal recessive inheritance model, including homozygous and compound heterozygous variants; (4) X-linked model; and (5) cosegregated mutations. After excluding the known epilepsy-associated genes (Wang et al., 2017), the genes with null mutations, *de novo* mutations, biallelic mutations, hemizygous mutations, and mutations with segregations were considered for further evaluation as possible novel epilepsy genes. Conservation of mutated positions was evaluated using sequence alignment of different species. All *RYR2* mutations were annotated based on the transcript NM_001035.2. Variants having potential clinical significance were confirmed by Sanger sequencing.

Protein modeling was performed to predict the effects of missense variants on molecular structure by using SWISS-MODEL (Shen et al., 2018)^3^, based on the updated template of 6jiy.1.A.pdb^4^. PyMOL 1.7 was used for three-dimensional protein structure visualization and analysis.

### Statistical Analysis

SPSS Statistics 26.0 was used for statistical analysis. The frequencies of the *RYR2* variants in the cohort of BECTS and in the control populations were compared by Fisher exact test (CONVERGE consortium, 2015). A *p*-value of < 0.05 was considered to be statistically significant.

### Evaluating *RYR2* Gene as a Novel Candidate Epilepsy Gene

To explore the genotype–phenotype association, we used the Clinical Validity Framework that was developed by Clinical Genome Resource (ClinGen) to evaluate *RYR2* as a novel candidate epilepsy gene (Strande et al., 2017).

## Results

### Identification of *RYR2* Mutations

**FIGURE 1:**
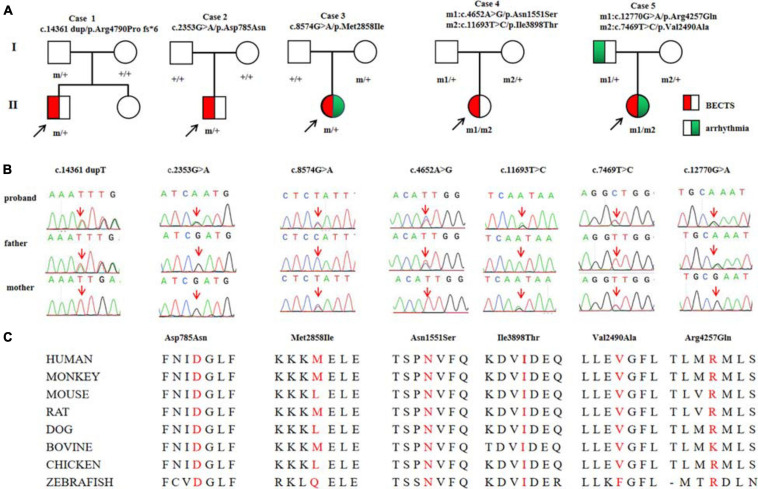
Genetic data on the patients with *RYR2* mutations. **(A)** Pedigrees of the five cases with *RYR2* mutations and their corresponding phenotypes. **(B)** DNA sequence chromatogram of the *RYR2* mutations. Arrows indicate the positions of the mutations. **(C)** The amino acid sequence alignment of the residues of missense mutations shows Asp785, Asn1551, and Ile3898 are highly conserved in various species, whereas Val2490, Met2858, and Arg4257 are less conserved.

Among the 292 patients with BECTS, *RYR2* mutations were identified in five unrelated cases. The mutations included one heterozygous frameshift mutation (c.14361dup/p.Arg4790Pro fs^∗^6), two heterozygous missense mutations (c.2353G > A/p.Asp785Asn and c.8574G > A/p.Met2858Ile), and two pairs of compound heterozygous mutations (c.4652A > G/p.Asn1551Ser and c.11693T > C/p.Ile3898Thr, c.7469T > C/p.Val2490Ala and c.12770G > A/p.Arg4257Gln, respectively; Figures 1A,B). The heterozygous frameshift mutation Arg4790Pro fs^∗^6 was inherited from his asymptomatic father. The heterozygous Asp785Asn was a *de novo* mutation. Segregation of the identified mutations is shown in Figure 1A.

Mutation Arg4790Pro fs^∗^6 was assumed to be deleterious by causing premature termination of transcript and leading to complete loss of RYR-2 function or haploinsufficiency. All the missense mutations were suggested to be damaging by at least three web-based prediction tools (Table 1). These mutations are not present or at low MAF in gnomAD database (the MAF of Asp785Asn and Asn1551Ser were 4.0e-6 and 3.0e-4, respectively). The missense mutations (Asp785Asn, Asn1551Ser, and Ile3898Thr) affected amino acid residues that are highly conserved in various species, whereas the missense mutations (Val2490Ala, Met2858Ile, and Arg4257Gln) were less conserved by sequence alignment (Figure 1C), but were predicted to be conserved by *in silico* tools (Table 1). All the five cases had no other pathogenic or likely pathogenic mutations in the 977 genes known to be associated with seizure disorders (Wang et al., 2017). A statistical analysis on the frequency of the *RYR2* variants showed significant difference between the present cohort and the control populations in gnomAD (7/584 vs. 40/108530 in controls of all populations, *p* = 6.34e-9; vs. 35/8638 in controls of East-Asian population, *p* = 0.015) (Table 2).

**TABLE 1:** Genetic features of the individuals with *RYR2* mutations.

Case	Position	NCBI SNP number	cDNA change (NM_001035.2)	Protein change	Inherit.	MAF	MAF-EAS	SIFT	PP2_Var	Mutation taster	CADD	GERP++	phyloP	phastCons	SiPhy
Case1	chr1:237972262	–	c.14361dup	p.Arg4790Pro fs*6	Paternal	–	–	–	–	–	–	–	–	–	–
Case 2	chr1:237664160	rs774486571	c.2353G > A	p.Asp785Asn	*De novo*	4.06e-6	0	D (0.036)	PD (0.957)	D (1.000)	D (23.9)	C (5.98)	C (9.994)	C (1.000)	C (20.458)
Case 3	chr1:237831242	–	c.8574G > A	p.Met2858Ile	Maternal	–	–	T (0.185)	B (0.012)	D (0.998)	D (23.1)	C (4.44)	C (4.702)	C (1.000)	C (15.336)
Case 4	chr1:237765380	rs185237690	c.4652A > G	p.Asn1551Ser	Paternal	3.0e-4	4.0e-3	T (0.295)	B (0.401)	D (0.983)	D (23.9)	C (5.53)	C (7.568)	C (1.000)	C (15.651)
	chr1:237936866	–	c.11693T > C	p.Ile3898Thr	Maternal	–	–	D (0.000)	PD (0.997)	D (1.000)	D (28.0)	C (5.13)	C (8.010)	C (1.000)	C (15.262)
Case 5	chr1:237811870	–	c.7469T > C	p.Val2490Ala	Maternal	–	–	T (0.404)	PD (0.996)	D (1.000)	D (23.7)	C (5.16)	C (8.017)	C (1.000)	C (15.285)
	chr1: 237947782	rs371396204	c.12770G > A	p.Arg4257Gln	Paternal	0	0	T (0.273)	B (0.079)	D (0.561)	T (12.69)	C (5.11)	C (4.146)	NC (0.999)	NC (6.101)

*B, benign; C, conserved; CADD, combined annotation dependent depletion; D, damaging; ExAC, Exome Aggregation Consortium; MAF, minor allele frequency from Genome Aggregation Database; MAF-EAS, minor allele frequency from Genome Aggregation Database–East Asia population; NC, non-conserved; P, polymorphism; PP2_Var, Polyphen2_HVAR; PD, probably damaging; T, tolerable.*

**TABLE 2:** Gene-based burden analysis for *RYR2* mutations identified in this study.

	Allele count/number in this study (%)	Allele count/number in controls of gnomAD-all populations (%)	Allele count/number in controls of gnomAD-East Asian populations (%)
**Identified *RYR2* mutations**			
c.14361dup/p.Arg4790Pro fs*6	1/584 (0.17)	−/−	−/−
c.2353G > A/p.Asp785Asn	1/584 (0.17)	1/108530 (0.00092)	0/8638 (0)
c.8574G > A/p.Met2858Ile	1/584 (0.17)	−/−	−/−
c.4652A > G/p.Asn1551Ser	1/584 (0.17)	39/119132 (0.0327)	35/9528 (0.367)
c.11693T > C/p.Ile3898Thr	1/584 (0.17)	−/−	−/−
c.7469T > C/p.Val2490Ala	1/584 (0.17)	−/−	−/−
c.12770G > A/p.Arg4257Gln	1/584 (0.17)	−/−	−/−
**Total**	7/584 (1.20)	40/108530 (0.0369)	35/8638 (0.4052)
***P-*value**		6.34e-9	0.015
**OR (95% CI)**		32.90 (14.68–73.76)	2.98 (1.32–6.74)

*P-values and odds ratio were estimated with two-sided Fisher exact test. CI, confidence interval; gnomAD, Genome Aggregation Database; OR, odds ratio.*

### Molecular Effect of *RYR2* Mutations

**FIGURE 2:**
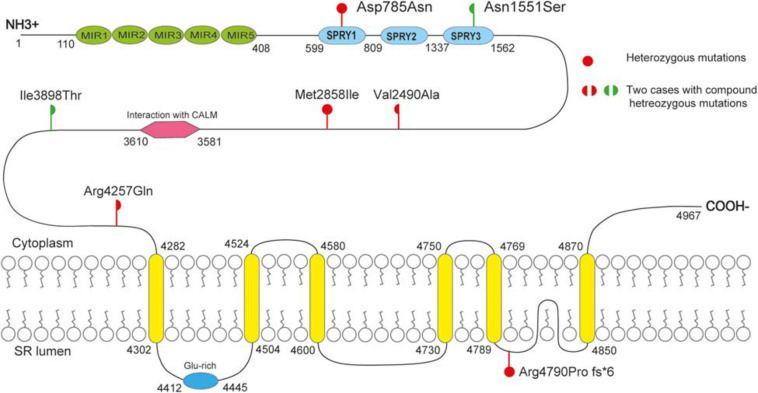
Schematic illustration of mutations. All the missense mutations were located in the cytoplasmic part of RYR-2 protein. The *de novo* missense mutation Asp785Asn was located in SPRY1 domain; mutation Asn551 was located in SPRY3 domain.

**FIGURE 3:**
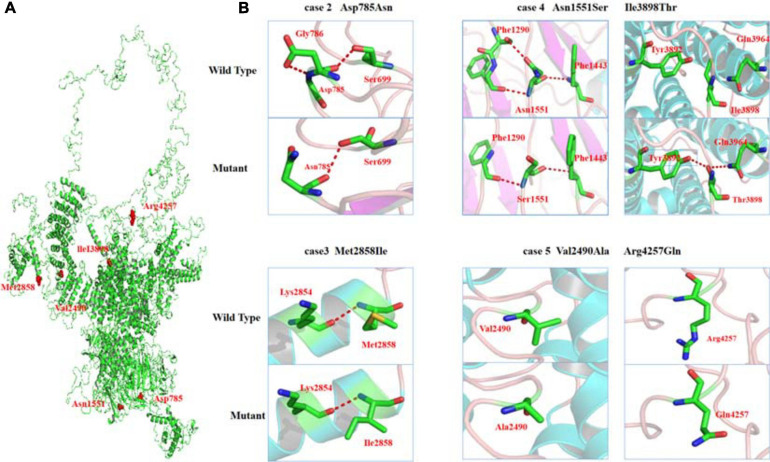
Crystal structure of RYR-2 (PDB: 6jiy) and hydrogen bond changes of the mutants. **(A)** Overview of RYR2 and positions of the mutations. **(B)** Asp785Asn, hydrogen bond with Gly786 disappeared; Asn1551Ser, hydrogen bond with Phe1290 disappeared; Ile3898Thr, formed new hydrogen bonds with Tyr3892 and Gln3964.

As shown schematically in Figure 2, the molecular length of RYR-2 protein includes 4,967 amino acids^5^. Topological domains (1–4,281) and (4,871–4,967) are located within the cytoplasm. Six helical domains (4,282–4,302, 4,504–4,524, 4,580–4,600, 4,730–4,750, 4,769–4,789, and 4,850–4,870) and one pore-forming domain (4,820–4,829) are transmembrane. The cytoplasmic parts include MIR1-5 domains (110–408), SPRY1-3 domains (599–809, 1,025–1,222, and 1,337–1,562), and a domain of interaction with CALM (3,581–3,610). All the missense mutations were located in the cytoplasmic part of RYR-2 protein. Asp785Asn was located in SPRY1 domain, and Asn1551Ser was located in SPRY3 domain (Figure 2). The molecular effect of the missense mutations was further analyzed by protein modeling using SWISS-MODEL. Three missense mutations changed the hydrogen bands. Residue Asp785 formed hydrogen bonds with Gly786 and Ser699 originally. When aspartic acid was replaced by asparagine at residue Asp785, the hydrogen bond with Gly786 was destroyed. Residue Asn1551 originally formed two hydrogen bonds with Phe1290 and one hydrogen bond with Phe1443. When asparagine was replaced by serine at residue Asn1551, one of the hydrogen bonds with Phe1290 disappeared. Residue Ile3898 had no hydrogen bond with surrounding residues. When isoleucine was replaced by threonine at residue Ile 3898, two new hydrogen bonds with Tyr3892 and Gln3964 were formed. The mutations Met2858Ile, Val2490Ala, and Arg4257Gln did not change the hydrogen bonds, of which Val2490Ala and Arg4257Gln were a pair of compound heterozygous mutation (Figure 3).

### Clinical Features of the Cases With *RYR2* Mutations

**FIGURE 4:**
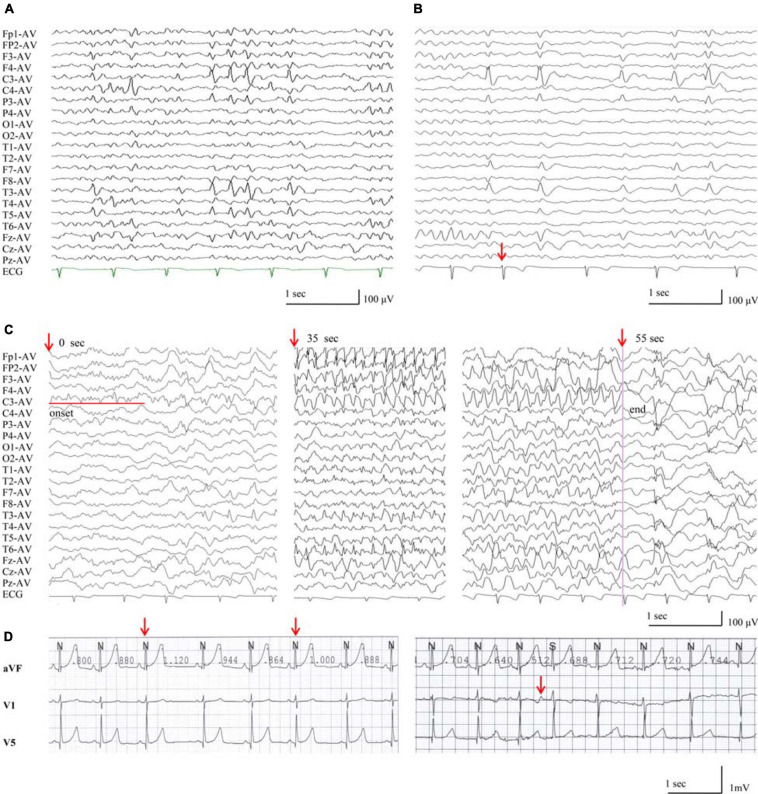
EEG discharges in the patients with *RYR2* mutations. **(A)** Interictal EEG of case 4 showed left or right centrotemporal spike and slow waves (obtained at the age of 11 years). **(B)** Interictal EEG of case 3, the red arrow indicates electrodes recording ECG of arrhythmia during NREMIII sleep (at the age of 7 years). **(C)** Ictal EEG of case 3, the red arrows indicate timing of seizure (at the age of 7 years). **(D)** The ECG of case 5 showed sinus arrhythmia and atrial premature beats.

All probands, including two boys and three girls, showed childhood-onset focal epilepsy (Figure 1A). Table 3 summarized the main clinical features of the cases with *RYR2* mutations. The onset age of seizures ranged from 4 to 12 years. All the cases had infrequent generalized tonic–clonic seizure (GTCS) and/or complex partial seizure. All the cases had normal development, intelligence, and speech ability. They did not have any other neurological symptoms, either. The EEGs showed unilateral or bilateral independent centrotemporal discharges, predominantly during sleep in all probands (representatives in Figures 4A,B). Arrhythmia was found in two families (three individuals in cases 3 and 5). In case 3, arrhythmias in interictal stage were recorded; her ictal EEG showed a seizure originated from the left central area, which was not secondary to cardiac attack (Figure 4C). The probands in case 5 had sinus arrhythmia with occasional atrial premature beats (Figure 4D). Her father presented the symptom of palpitations. His ECG examination also showed sinus arrhythmia. Additionally, light tricuspid regurgitation was detected in case 4, and light tricuspid and pulmonary valve regurgitations were detected in case 5 by cardiac ultrasound. The brain MRI scans in all patients were normal. All the cases got seizure-free with monotherapy of valproate or lamotrigine, or combination of valproate and levetiracetam.

**TABLE 3:** Clinical features of the individuals with *RYR2* mutations.

Case	Variants (NM_001035.2)	Gender	Age	Seizure onset	Seizure and frequency	Seizure timing	EEG	ECG	CUS	Effective AEDs	Seizure-free duration (years)
Case 1	p.Arg4790Pro fs*6	Male	10 years	8 years	sGTCS, 6 times/years	Nocturnal	Right frontal and bilateral central sharp waves and sharp-slow waves	Normal	NA	VPA	1.5
Case 2	p.Asp785Asn	Male	18 years	12 years, 5 months	GTCS, 2 times/months	Nocturnal	Bilateral centrotemporal sharp-slow waves	Normal	normal	VPA, LEV	5
Case 3	p.Met2858Ile	Female	14 years	6 years, 3 months	CPS and sGTCS, 2–4 times/months	Nocturnal	Ictal: sGTCS originated from left central region; interictal: bilateral centroemporal and parietal spike or sharp waves	Sinus arrhythmia	NA	VPA, LEV	6
Case 4	p.Asn1551Ser, p.Ile3898Thr	Female	13 years	4 years	CPS and GTCS, once/month	Diurnal and nocturnal	Bilateral centrotemporal spikes, sharp waves, and sharp-slow waves	Normal	Light tricuspid and pulmonary valve regurgitation	VPA	7
Case 5	p.Val2490Ala, p.Arg4257Gln	Female	7 years	5 years	sGTCS, 1–2 times/months	Nocturnal	Bilateral centrotemporal spikes	Sinus arrhythmia and occasional atrial premature	Light tricuspid regurgitation	LTG	1

*AEDs, antiepileptic drugs; CPS, complex partial seizure; EEG, electroencephalogram; CUS, cardiac ultrasound; ECG, electrocardiograph; GTCS, generalized tonic–clonic seizure; sGTCS, secondary generalized tonic–clonic seizure; LEV, levetiracetam; LTG, lamotrigine; NA, not available; VPA, valproate.*

### Evaluation of Epilepsy as a Novel Phenotype of *RYR2* Variants

We evaluated the *RYR2* variants-epilepsy correlation using ClinGen Clinical-Validity Framework. Both total allowable points for genetic aspect and experimental aspect were 6 points. The results of clinical validity summary matrix were 12 points that was categorized as “strong,” supporting the association between *RYR2* variants and epilepsy (Table 4).

**TABLE 4:** Evaluating the clinical validity of *RYR2*-epilepsy associations based on the framework developed by the clinical genome resource.

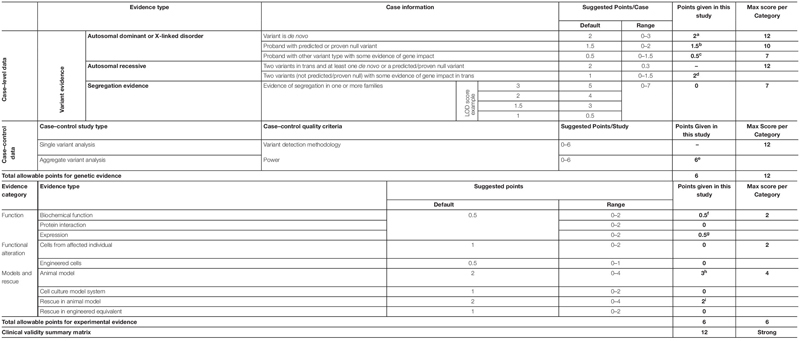

*^*a*^One de novo missense variant (Case 2: c.2353G > A/p.Asp785Asn) (2 points/case × 1 cases).*

*^*b*^One proband with predicted or proven null variant (case 1: c.14361dup/p.Arg4790Pro fs^∗^6) (1.5 points/case × 1 cases).*

*^*c*^One variant (case 3: c.8574G > A/p.Met2858Ile) did not appear in general populations in gnomAD database (0.1 points/case × 1 cases). It was predicted damaging by multiple in silico programs (0.4 points/case × 1 cases).*

*^*d*^Two compound variants with evidence of gene impact in trans (1 point/case × 2 cases).*

*^*e*^This is an aggregate analysis. Comparing to allele number in gnomAD-control populations, the statistical test showed OR of all variants in aggregate is 32.90 [confidence interval (CI), 14.68–73.76; P = 6.34 × 10^–9^]. If matched by ethnicity, OR of all variants in aggregate is 2.98 (CI, 1.32–6.74; P = 0.015) (assigned 6 points). The points are not included in total allowable points for genetic evidence.*

*^*f*^RYR2 encodes a ryanodine receptor found in sarcoplasmic reticulum. The encoded protein is one of the components of a calcium channel, composed of a tetramer of the ryanodine receptor proteins and a tetramer of FK506 binding protein 1B proteins. The RYR-2 is required for excitation–contraction coupling in the heart. It is also present in the central nervous system and contributes to vesicular transmitter release and postsynaptic dendritic spine function (assigned 0.5 points).*

*^*g*^The RYR-2 protein is ubiquitously distributed and highly expressed in the heart and brain (assigned 0.5 points).*

*^*h*^Homozygous RYR2 null mice show embryonic lethality during organogenesis (MGI: 99685). A human leaky Ryr2 mutation, R176Q (RQ), alters neurotransmitter release probability in mice and significantly lowers the threshold for spreading depolarization in dorsal medulla, leading to cardiorespiratory collapse. Rare episodes of sinus bradycardia, spontaneous seizure, and sudden death were detected in RQ/ + mutant mice in vivo (Aiba et al., 2016;). The heterozygous mice for the R2474S mutation in Ryr2 (Ryr2-R2474S mice) exhibited spontaneous generalized tonic–clonic seizures (Lehnart et al., 2008;) (assigned 3 points).*

*^*i*^Treatment with a novel RyR2-specific compound (S107) that enhances the binding of calstabin2 to the mutant Ryr2-R2474S channel inhibited the channel leak and prevented cardiac arrhythmias and raised the seizure threshold (Lehnart et al., 2008;) (assigned 2 points).*

## Discussion

In the present study, *RYR2* mutations were identified in five unrelated cases with BECTS, including one case with heterozygous frameshift mutation, one case with *de novo* heterozygous missense mutation, and two cases with compound heterozygous mutations. As a subunit of homotetramer, RYR-2 constitutes the calcium channel that regulates intracellular calcium concentration (Marx et al., 2000). The RYR-2 protein is mainly distributed on the ER membrane and is ubiquitously expressed with predominance in the heart and brain during all stages of human development^6^. In *RYR2*-deficiency mice, homozygotes showed embryonic lethality during organogenesis and altered cardiomyocyte morphology (Choy et al., 2016), suggesting that *RYR2* is vital for fetal development. The heterozygous deficiency mice presented increased startle reflex, as well as cardiovascular abnormities, such as abnormal myocardial fiber physiology, irregular heartbeat, and ventricular premature beat (Loaiza et al., 2013; Liu et al., 2014; Choy et al., 2016; Dickinson et al., 2016). The *RYR2* mutations have been established in the etiology of ARVD2 and CPVT (Tiso et al., 2002). RYR-2 mediates intracellular Ca^2+^ release to trigger cardiomyocyte contraction through L-type voltage gated channels. Abnormal functioning of RYR-2 will result in imbalance of intracellular calcium ion homeostasis, leading to arrhythmia, cardiac hypertrophy, heart failure, and other heart abnormalities (Seidel et al., 2020). Patients with ARVD2 or CPVT occasionally had seizures. However, the relationship between *RYR2* and epilepsy is unclear. In the present study, *RYR2* mutations were identified in five cases with BECTS. The frequency of the *RYR2* variants in the cohort of epilepsy was significantly higher than that in control populations in gnomAD. Taken together the facts that RYR-2 is highly expressed in the brain (Martin et al., 1998) and that *RYR2* knockout animals presented increased cellular excitability (Loaiza et al., 2013; Liu et al., 2014; Choy et al., 2016; Dickinson et al., 2016; Seidel et al., 2020), it is considered that *RYR2* gene is potentially a candidate pathogenic gene of epilepsy. The evidence from ClinGen Clinical-Validity Framework also supports a strong association between *RYR2* mutations and epilepsy.

Generally, calcium homeostasis is critical for neuronal stability, and RYR-2 plays a key role in the regulation of calcium homeostasis (Lin et al., 2018). Intracellular Ca^2+^ is an important secondary messenger for signal transduction and essential for cellular processes such as excitatory contraction coupling. The major sources of intracellular Ca^2+^ include the sarcoplasmic reticulum (SR) in striated muscle and the ER in other cell types (Otsu et al., 1990). There are two major Ca^2+^ release channels localized in the SR/ER, the ryanodine receptors (RyRs) (Lanner et al., 2010), and inositol 1,4,5-triphosphate receptors (IP3Rs) (Nixon et al., 1994). RyRs, encoded by *RYR1*, *RYR2*, and *RYR3*, are widely distributed in the central nervous system of human beings; RYR-2 is the most abundant isoform in the brain, especially in hippocampus (Martin et al., 1998), suggesting a central role in Ca^2+^ signaling and Ca^2+^ homeostasis in the human central nervous system. Previous studies in animals have indicated that the *RYR2* deficiency is associated with epilepsy or alterations of electrical conduction in nerve cells. GTCSs were observed in knock-in mouse model of heterozygous *RYR2* missense mutation Arg2474Ser, which was identified in the patients with CPVT (Lehnart et al., 2008). *RYR2* Arg176Gln mutant mice altered neurotransmitter releasing probability and lowered the threshold for spreading depolarization significantly, leading to spreading depolarization across the neocortex and brainstem dorsal medulla autonomic microcircuits (Aiba et al., 2016). Clinically, in a family with *RYR2* mutation (c.229G > A/p.Ala77Thr) that caused CPVT, one affected individual presented unprovoked generalized seizures and epileptiform activities (Yap and Smyth, 2019). A compound heterozygous missense mutation (c.3248A > G/p.Glu1083Gly and c. 6779 C > T/p.Arg2260Leu) had been identified in a child with early infantile epileptic encephalopathy (Peng et al., 2018). The ictal EEG with focal originated discharges presented in this study provided direct evidence of epileptic seizure, instead of attacks due to heart abnormalities. These evidences suggest *RYR2* mutations were potentially associated with epilepsy. All cases in this study were diagnosed as BECTS, suggesting *RYR2* was potentially a causative gene of BECTS.

Recent studies have shown close relationships between epilepsy and heart disorders that were caused by several genetic abnormalities (Parisi et al., 2013; Partemi et al., 2015; Coll et al., 2016, 2017). These abnormalities were also associated with sudden unexpected death in epilepsy (SUDEP), which is the leading cause of epilepsy-related premature mortality. Three individuals with *RYR2* mutations in the present study showed arrhythmia. The two probands with compound heterozygous missense mutations presented mild cardiac structural abnormalities. The *RYR2* mutations have been established in the etiology of ARVD2 and CPVT (Tiso et al., 2002), in which arrhythmia was common. So far, 547 *RYR2* mutations have been identified, mostly in patients with cardiac disorders (HGMD)^7^. A recent study showed that one *RYR2* mutation was associated with SUDEP, and the patient had occasionally nocturnal seizures (Bagnall et al., 2016). Risk of triggering cardiac arrhythmias by AEDs has also been reported (Shmuely et al., 2017; Yap and Smyth, 2019; Banfi et al., 2020). Thus, special attention should be paid to the patients with *RYR2* mutations, who may present benign epilepsy such as BECTS but potentially require careful follow-up and specialized health consultation due to the coexistence of epilepsy and heart disease.

This study has several limitations. The direct functional effects of the mutations identified in the present study were not examined. The mutations associated with heart disorders presented functional abnormalities of loss of function or gain of function. Further studies are required to characterize the functional effects of the mutations associated with epilepsy. In the present study, two pairs of compound heterozygous mutations (p.Asn1551Ser and p.Ile3898Thr, and p.Val2490Ala and p.Arg4257Gln, respectively) were identified in the patients with epilepsy. *RYR2* is usually regarded as a dominant gene. Biallelic pathogenic variants have been reported in dominant genes, such as *GRIN1* gene (Lemke et al., 2016). Generally, gene variants differ in damage effect, which varies from mild to severe, depending on factors such as the molecular subregional effect as shown in our recent study (Tang et al., 2020). It is possible that these *RYR2* mutations were of less damage impact. Each of them alone seemed not pathogenic, whereas biallelic variants compounding together would be pathogenic. Two individuals, the father with p.Arg4790Profs^∗^6 and the mother with p.Met2858Ile, were asymptomatic, suggesting incomplete penetrance. The patients with *RYR2* mutations presented BECTS, which was generally a mild form of epilepsy, reflecting relatively weak pathogenicity of the *RYR2*

mutations and potentially explaining the incomplete penetrance. However, it is unknown whether *RYR2* mutations were associated with more severe forms of epilepsy, and the whole spectrum of phenotype of *RYR2* mutations warrants further studies. Several patients presented cardiac abnormalities. The occurrence and significance of cardiac phenotype in epilepsy patients with *RYR2* mutations and their long-term outcome need further observation and follow-up.

## Conclusion

In conclusion, we identified *RYR2* mutations in five unrelated cases with BECTS. The genetic and molecular evidence implied that the mutations led to the defect of the *RYR2* gene. Taken together the data from gene expression profile and *RYR2* deficiency animal model, it is suggested that *RYR2* was potentially a causative gene epilepsy. Special attention should be paid to the patients with *RYR2* mutations due to the coexistence of epilepsy and heart disease, which potentially were potentially associated with SUDEP.

## Data Availability Statement

The original contributions presented in the study are included in the article/supplementary material, further inquiries can be directed to the corresponding authors.

## Ethics Statement

The studies involving human participants were reviewed and approved by the Ethics Committee of the Second Affiliated Hospital of Guangzhou Medical University. Written informed consent to participate in this study was provided by the participants’ legal guardian/next of kin.

## Author Contributions

Y-HY and W-PL designed and conceptualized the study. M-GM, X-RL, JW, B-ML, BL, and D-TL collected and analyzed the data. M-GM and X-RL drafted the manuscript for intellectual content. M-GM, X-RL, YW, Y-WS, TS, Y-HY, and W-PL revised the manuscript for intellectual content. BL and D-TL provided software support for 3D structure model. M-GM and D-TL prepared the figures. All authors have read and approved the final draft of the manuscript.

## Conflict of Interest

The authors declare that the research was conducted in the absence of any commercial or financial relationships that could be construed as a potential conflict of interest.

## Footnotes

1 http://www.omim.org

2 http://varcards.biols.ac.cn/

3 https://swissmodel.expasy.org/

4 http://www.rcsb.org

5 https://www.uniprot.org

6 http://www.gdap.org.cn/geneinfo.php

7 http://www.hgmd.cf.ac.uk/ac/search.php
